# Anxiety in patients with ankylosing spondylitis in southern-Tunisia: Level and associated factors

**DOI:** 10.1192/j.eurpsy.2024.1010

**Published:** 2024-08-27

**Authors:** A. Feki, I. Sellami, N. Ketata, M. Baklouti, Z. Gassara, S. Ben Djemaa, S. Ben Djemaa, M. Ezzeddine, M. H. Kallel, H. Fourati, R. Akrout, Y. Mejdoub, S. Baklouti

**Affiliations:** ^1^Rheumatology; ^2^Occupational medicine, Hedi Chaker Hospital; ^3^ Medicine university; ^4^Preventive medicine, Hedi Chaker Hospital, Sfax, Tunisia

## Abstract

**Introduction:**

Ankylosing spondylitis (AS) is the second most common rheumatic disease after rheumatoid arthritis. The significant functional impact of this chronic disease can affect patients’ mental health.

**Objectives:**

The aim of this study was to determine the prevalence of anxiety in subjects with AS in Southern-Tunisia and to identify its associated factors.

**Methods:**

It was a retrospective study conducted in 2021 over a period of 5 years on patients with AS consulting the rheumatology department at the Hedi Chaker University Hospital in SFAX, Southern-Tunisia. The “Anxiety and Depression scale” was used to screen for anxiety. A score ≥11 defined confirmed anxiety symptoms.

**Results:**

Of the 62 patients, 35 were male (56.5%), giving a male to female ratio of 1.3. Twenty-seven patients (43.5%) were aged between 35 and 50 years. The level of education was primary in 19 cases (30.6%) and university in 15 cases (24.2%). A family history of chronic disease was present in 32 cases (51.6%). Severe fatigue was noted among 27 patients (43.5%). Quality of life was poor in 39 patients (62.9%). The mean anxiety score was 11.35±4.6. Thirty-four subjects (54.8%) had confirmed anxiety symptoms and 19 (30.5%) had borderline symptoms. Confirmed anxiety was significantly associated with the educational level (p=0.03) (illiterate: 87.5%, primary: 68.4%, secondary: 35% and university: 46.7%). Similarly, having a family history of chronic disease (OR=3.3; p=0.02), suffering from severe fatigue (OR=36, p<0.01), having associated depression (HAD score≥11) (OR=19.5; p<0.001) and having poor quality of life [Ankylosing spondylitis quality of life questionnaire (Asqol) Score ≥13] (OR=15.8; p<0.001) were statistically associated with higher prevalence of confirmed anxiety symptoms.

**Conclusions:**

It was found that patients treated for AS frequently suffer from psychological co-morbidities, particularly anxiety, which can lead to a further deterioration in their quality of life and even their withdrawal from active life. Thus, anxiety should not be ignored when treating these patients.

**Disclosure of Interest:**

None Declared

